# PDE9 Inhibitor PF-04447943 Attenuates DSS-Induced Colitis by Suppressing Oxidative Stress, Inflammation, and Regulating T-Cell Polarization

**DOI:** 10.3389/fphar.2021.643215

**Published:** 2021-04-08

**Authors:** Mohammad Nasiruddin Rana, Jie Lu, Enfu Xue, Jingjing Ruan, Yuting Liu, Lejun Zhang, Rana Dhar, Yajun Li, Zhengqiang Hu, Jie Zhou, Wangqian Ma, Huifang Tang

**Affiliations:** ^1^ Department of Pharmacology, School of Basic Medical Sciences, Zhejiang University, Hangzhou, China; ^2^ Clinical Laboratory, Sir Run Run Shaw Hospital, School of Medicine, Zhejiang University, Hangzhou, China; ^3^ Department of Anesthesiology, Tongde Hospital of Zhejiang Province, Hangzhou, China; ^4^ Department of Gastroenterology, Second Affiliated Hospital, Zhejiang University College of Medicine, Hangzhou, China

**Keywords:** ulcerative colitis, PF-04447943, inflammasome, Treg/Th17 cell balance, PDE9A, oxidative stress

## Abstract

Ulcerative colitis (UC) is a form of inflammatory bowel disease, which manifests as irritation or swelling and sores in the large intestine in a relapsing and remitting manner. In a dextran sulfate sodium sulfate (DSS)-induced UC model in female mice, we found that the levels of cyclic guanosine monophosphate (cGMP) are reduced, while the expression of phosphodiesterase 9A (PDE9A) is highest among all phosphodiesterase (PDEs). Since PDE9 has the highest affinity toward cGMP, we evaluated the selective PDE9 inhibitor PF-04447943 (PF) as a potential candidate for UC treatment. PF has been extensively studies in cognitive function and in sickle cell disease, but not in models for inflammatory bowel disease (IBD). Therefore, we used female C57BL/6 mice treated with 3% DSS alone or co-treated with PF or sulfasalazine (SASP) to study the body weight, colon length, histopathology, and measure superoxide dismutase (SOD), malondialdehyde (MDA), and cGMP level, as well as cytokines such as tumor necrosis factor-alpha (TNF-α), interleukin-6 (IL-6), interleukin-17 (IL-17), interleukin-12/23 (IL-12/23), interleukin-10 (IL-10), and pathways including nuclear factor kappa B (NF-κB), signal transducer and activator of transcription 3 (STAT3), and inflammasome activation. In addition, the number of dendritic cells (DC) and regulatory T cells (Treg cell) was assessed in the spleen, lymph node, and colon using flow cytometry. DSS reduced the number of goblet cells, decreased colon lengths and body weights, all of them were attenuated by PF treatment. It also suppressed the elevated level of inflammatory cytokines and increased level the anti-inflammatory cytokine, IL-10. PF treatment also reduced the DSS-induced inflammation by suppressing oxidative stress, NF-κB, STAT3, and inflammasome activation, by upregulating nuclear factor erythroid 2-related factor 2 (Nrf-2) and its downstream proteins via extracellular signal-regulated kinase (ERK) phosphorylation. Importantly, PF reversed imbalance in Treg/T helper 17 cells (Th17) cells ratio, possibly by regulating dendritic cells and Treg developmental process. In summary, this study shows the protective effect of a PDE9A inhibitor in ulcerative colitis by suppressing oxidative stress and inflammation as well as reversing the Treg/Th17 cells imbalance.

## Introduction

Inflammatory bowel disease (IBD) is an idiopathic inflammatory disorder of the gastrointestinal tract, characterized by the relapsing and remitting disease with dysregulated mucosal immunity (Okamoto et al., 2020). Ulcerative colitis (UC) and Crohn's diseases (CD) are two forms of IBD, which are closely associated with genetics, lifestyle, and environmental etiological factors (Ng et al., 2013; Ng et al., 2015). For decades, the rate of incidence and prevalence of IBD has increased globally which puts the patients in the economical and psychological stress as chronic IBD harbor colorectal cancer (Bernstein, 2017). For the United States alone, almost three million people have been reported in 2015 with hospitalization cost of $11,345 and $13,412 per patient for Crohn’s disease and ulcerative colitis, respectively, (Centers for Disease control and Prevention, 2020).

The pathogenesis of IBD is multifactorial and yet to be fully understood. Both inflammation and oxidative stress are thought to be implicated as key mechanisms of the pathogenesis of UC. Elevation of tumor necrosis factor alpha (TNF-α) increases interleukin 1 beta (IL-1β), interleukin 6 (IL-6), and interleukin 33 (IL-33) expression, of which IL-6 activates the signal transducer and activator of transcription 3 (STAT3) to initiate strong inflammatory response both in UC and CD (Lee et al., 2018). Alongside this, the balance between anti-inflammatory Treg cells and pro-inflammatory Th17 cells also plays a pivotal role to protect the integrity of mucosal immunity. In UC, specific cytokines (e.g., IL-6), and transcription factors (e.g., NF-κB, retinoic acid-related orphan receptor gamma t (RORγt), STAT3 are indispensable for differentiation and proliferation of Th-17 cells. Therefore, these cytokines and transcription factor serves as potential therapeutic targets (Brand, 2009). Moreover, the activated T-cells, macrophages, and antigen-presenting cells (APC) release reactive oxygen species (ROS) and pro-inflammatory cytokines. Hence, dysbiosis and ROS exacerbations are closely interrelated (Irrazábal et al., 2014). The augmented ROS level orchestrates the oxidative stress, compromising the function of tight junctions, the integrity of the epithelial cells and butyrate oxidation (β-oxidation) for healthy colonic epithelial cells (Novak and Mollen, 2015; Novak and Mollen, 2015; Mitchell and Maxson, 2016; Shin and Kim, 2018). Several classes of therapeutic agents including 5-aminosalicylate, corticosteroids, thiopurines, anti-TNF-α, anti-integrins, and anti-c-Jun N-terminal kinase (JNK) therapies have been evaluated and used to manage the UC (Tripathi and Feuerstein, 2019). Unfortunately, these agents only address the symptoms but do not cure the disease (Rubin et al., 2019). Moreover, several side effects like anemia, kidney and liver dysfunction, cataracts, osteoporosis, leukopenia, malignancy, immunosuppression, and increased risk for opportunistic infection have been recorded, with some of these being irreversible (Tripathi and Feuerstein, 2019). Therefore, finding the novel therapeutics to cure UC without side effect or with limited side effects is urgent.

Cyclic nucleotide phosphodiesterase (PDE) inhibitors had shown their remarkable outcomes in different inflammation associated disorders (Peixoto et al., 2017; Li et al., 2018). The PDE superfamily comprises 11 members (PDE1-11), the common N-terminus to C-terminus structure is profoundly known for their catalytic action on cAMP and cGMP. The N-terminus of the PDE serves as the regulatory region which controls the function of the respective group, while C-terminus recognizes cAMP or cGMP or both as their substrate (Salter and Wierzbicki, 2007; Azevedo et al., 2014; Jansen et al., 2016). Among PDEs, PDE4, and PDE7 are selective to hydrolyze cAMP to 5′-AMP, while PDE5, PDE6, and PDE9 hydrolyze cGMP to 5′-GMP. In clinical UC patients, mucosal biopsies had shown the downregulation of guanylate cyclase-C (GC-C), its ligand guanylin and uroguanylin, which involved in the aggravation of the clinical condition. Therefore, the GC-C signaling pathway maybe involved in the progression of UC (Lan et al., 2016). Futhermore, experimental studies suggested that PDE5 inhibitor tadalafil and sildenafil are capable to retard UC. The mechanism involves in the suppression of inflammatory cytokines, oxidative stress, apoptosis, and improving the intestinal barrier function (Islam et al., 2017; Labib et al., 2017). Since PDE9 has the similar characteristic of PDE5, with the higher affinity for cGMP (Fisher et al., 1998), meanwhile, our previous study had shown that DSS induces the highest expression of PDE9 among the PDE superfamily (Supplementary Figure S1) So, we speculated that PDE9 inhibitor could also be a good candidate to attenuate UC.

PF-04447943 (PF) is a selective inhibitor of PDE9A with an IC_50_ value of 12 nM (Kleiman et al., 2012). Recently, PF is widely used to do the clinical trials for the neuropsychological and sickle cell disease. In the early studies, PF attenuates a scopolamine: induced cognitive deficit, and improve synaptic plasticity in neuropsychological disease animal model (Hutson et al., 2011; Vardigan et al., 2011), and reduces the leukocyte-platelet aggregation and soluble E-selectin activation in the sickle cell disease mice model (Jasuja et al., 2016). At the same time, PF has good pharmacokinetic properties, such as widely distribution and rapid absorption (1.9 h) (Charnigo et al., 2019b). The clinical studies suggested that PF is well tolerated in patients with stable sickle cell disease (Charnigo et al., 2019a) or Alzheimer disease (Schwam et al., 2014). So, it helped us to study its effect on UC model.

The study was aimed to evaluate the effect of PF-04447943 against DSS-induced colitis mice model and investigate the mechanism of action of PF-04447943. In this study, mice were treated with 3% DSS via drinking water for 7 days, PF-04447943 was administrated at different doses by oral gavage for the same time. Then, we evaluated the body weight change, the histopathology, oxidative stress, inflammation, inflammasome, autophagy, dendritic cell (DC) infiltration, Treg/Th17 cells balance, and related signaling pathway. Our current findings firstly suggested that PF-04447943 attenuates the DSS-induced colitis by suppressing oxidative stress, inflammation, inflammasome and the Treg/Th17 cells balance, and the mechanisms involved in regulating NF-kB, STAT3, Nrf2, and MAPK (ERK) pathway.

## Materials and Methods

### Chemicals and Reagents

PDE9A inhibitors PF-0444794 (Cat. No: MB3418) and sulfasalazine (SASP, Cat. No: MB5634) were purchased from Dalian Meilun Biotech Co., Ltd. (http://www.meilune.com/, China). Dextran sodium sulfate (DSS) (Cat. No. 160110) was purchased from MP Biomedicals (Canada). Malondialdehyde (MDA) assay kit (Cat. No: A003), total superoxide dismutase (SOD) assay kit (Cat. No: A001) were purchased from Nanjing Jianjian Technology Co., Ltd. (China). cGMP assay kit (Cat. No: HLE203555) was bought from Haling Bio (Shanghai, China). AB/PAS staining kit (Cat.No: GP1041) was purchased from Servicebio (Wuhan, China). TNF-α (Cat. No: DY410), IL-6 (Cat. No: DY406), IL-12/IL-23 (Cat. No: DY499), IL-17 (Cat. No: DY421), and IL-10 (Cat. No: DY417) were purchased from R& D systems (Minneapolis, MN, USA). PE anti-mouse/human CD11b (Cat. No: 101207), APC anti-mouse CD11c (Cat. No: 117309), APC anti-mouse CD25 (Cat. No: 102011), FITC anti-mouse I-Ad (Cat. No: 115005), PE anti-mouse FOXP3 (Cat. No: 320007), FITC anti-mouse CD4 (Cat. No: 100509) were purchased from BioLegend (San Diego, United States). Triisopropylethanesulfonyl (Tris) (Cat. No: 0497–500G) and sodium dodecyl sulfonate (SDS) (Cat. No: 0227–100G) were purchased from AMRESCO (United States).

### Animal

Female C57BL/6 mice (8 weeks, *N* = 60) were purchased from SLAC Laboratory Animal (Certificate No: SCXK 2017–0016, Shanghai, China). The mice (*N* = 60) were randomly divided into two batches, each of them had 30 mice. The first batch (*N* = 30) were assigned into six groups as mentioned in the experimental design for the study. The second batch (*N* = 30) was similarly divided into six groups to do flow cytometry. Here, the number of animals was calculated using “resource equation” approach for one-way ANOVA analysis (Arifin and Zahiruddin, 2017). Mice were housed in the animal center with the standard condition (23 ± 2°C, humidity, 55 ± 5%, and 12−12 h light/dark cycle). Mice were acclimatized for 1 week with autoclaved water and food ad libitum. The experimental protocols were approved by the Animal Ethics Committee of Zhejiang University (Approval number: 19277) in accordance with international guidelines.

### Preparation of Acute Colitis Mice Model and Experimental Design

In this study, mice were randomly divided into six groups as follows: 1) Control group (deionized water + deionized water, oral gavage, once daily); 2) DSS group (3% DSS + deionized water); 3) PF 3 group (3% DSS + PF 3 mg/kg); 4) PF 10 group (3% DSS + PF 10 mg/kg); 5) PF 30 group (3% DSS + PF 30 mg/kg); 6) SASP group (3% DSS + SASP 0.5 g/kg). To induce colitis, mice were allowed to drink 3% DSS (W/V) prepared with deionized water, and the control groups were treated with deionized water, PF-0444794 or SASP were administrated by oral gavage, once per day for 7 days. During the treatment period, the mice’s body weight was recorded daily. On day 7, mice were anesthetized with 1% pentobarbital sodium and sacrificed by collecting blood from the left eye. The colons, mesenteric lymph nodes, and spleen were collected. The length of the colon was measured and divided into three sections. Descending colon with rectum was fixed in 4% paraformaldehyde, and the remaining specimens were stored at −80°C.

### Periodic Acid Schiff Staining

A segment of the distal colon (0.5 cm) was immersed in 4% formaldehyde for 3 days and embedded in paraffin following standard procedure. Tissue was sectioned into 4 μM using Leica Biosystem (Germany). The tissue section was deparaffinized in xylene, rehydrated in a series of graded alcohol and water. Thereafter, the section was incubated in 0.5% periodic acid at room temperature (RT) for 15 min, followed by washing. The section was stained with Schiff's reagent at RT for 30 min and wash with warm water (Machorru-Rojas et al., 2019). Thereafter, the section was investigated and photographed under a light microscope (Model: BX51, Olympus, Japan; camera: DP20).

### Measurement of MDA, SOD, and cGMP Level in Colon

To detect the level and activity, colon tissue was minced and homogenized. The supernatant was then used to detect the level and activity of cGMP, MDA, and SOD, respectively, following the manufacturer’s instruction. cGMP was determined by ELISA, MDA, and SOD were determined by colorimetric assay.

### Measurement of Cytokines in Colon

To quantify the level of cytokines associated with inflammation and Th17 cell, TNF-α, IL-6, IL-10, IL-17, and IL-12/23 level in colon tissue were measured with the ELISA kit following the supplied protocol of the manufacturer. The colon tissue was minced into small pieces and homogenized in ice-cold PBS. The suspension was then centrifuged at 13,000 × g, 15 min, 4°C. The resultant supernatant was collected and stored at −80°C for ELISA assay. To prepare plate, the capture antibody was diluted to working concentration and used to coat a 96 well plate with 100 μl per well. The plate was sealed with a parafilm and incubated at room temperature (RT) for overnight. The detection antibody was aspirated and washed 3 times with phosphate buffered saline (PBS)-Tween (T) (PBS-T, 10 mM phosphate buffer pH 7.4, 150 mM NaCl, 0.05% Tween-20). The remaining droplets were removed by patting the plate on a paper towel. Block the plate with 150 μl of 2% BSA (Bovine serum albumin) for 2 h at RT. The plate was aspirated, washed for three times and patted on paper towel. 100 μl of sample/standard was added to each well and incubated for 2 h at RT. Following incubation, the aspiration step was repeated. The wells were then filled with 100 μl of diluted detection antibody and incubated for 2 h at RT. Following aspiration, 100 μl of the dilution of streptavidin-HRP (1:20) was added to each well and incubated in a dark place at RT for 20 min, followed by aspiration. The substrate solution (100 µl) was added and incubated in the dark place as before till color development (6–12 min). To stop the color development, 50 μl of stop solution was then added to each well and read the absorbance at 450 nm using microplate reader.

### Western Blot Analysis

Proteins from the colon and spleen were extracted by homogenization with cold RIPA (Radio Immunoprecipitation Assay) lysis buffer containing PMSF (Sigma, Missouri, United States), PhosSTOP and protease inhibitor cocktail (Roche, Mannheim, Germany). Following centrifugation (13,000 × g, 15 min, 4°C), the supernatant was collected and protein concentration was measured using Bio-rad reagent (Bio-Rad Inc. California, United States). The protein sample was then separated at 70 and 130 V for 45 min and 1 h, accordingly. This separated protein was transferred to nitrocellulose membranes (NC) membranes (BioTrace NT membrane, Gelman laboratory, Ann Arbor, Michigan, United States) for 90 min at 300 mA (Mini-protein II system, Bio-Rad). Thereafter, the NC membrane was then blocked for 1 h at RT with 5% bovine serum albumin (BSA) in Tris buffer saline (TBS) (20 mM Tris-HCl, pH 7.5, 500 mM NaCl). The membrane was incubated with primary antibody: Anti-*p*-ERK (1:1,000, ab76165, Abcam, United States), anti-ERK (1:3,000, ab79853, Abcam, United States), anti-*p*-AKT(Ser473) (1:1,000, D9E, #4060, CST, United States), anti-AKT (1:1,000, db1607, Diagbio, China), anti-*p*-STAT3 (Y705) (1:1,000, BS4181, Bioworld, China), anti-STAT3(79D7) (1:2000, #4904, CST, United States), anti-SOCS3 (1:1,000, #2923, CST, United States), anti-*p*-P65(S276) (1:1,000, BS4135, Bioworld, United States), anti-IKKα+β (s180 + s181) (1:500, ab55341, Abcam, United States), anti-NLRP3 (1:1,000, G-20B-0014, Adipogen Life Sciences, United States), anti-Caspase-1 (1:1,000, Ab179515, Abcam, United States), anti-ASC (1:1,000, F-9:sc-271054 Santa Cruz Bio Tech. United Stated), anti-LC3 (1:1,000, db760, Diagbio, China), anti-Nrf-2 (1:1,000, db3180, Diagbio, China), anti-NQO1 (1:1,000, 11451-1-AP, Proteintech, United States), anti-HO-1(1:1,000, db 4,329, digbio, China), anti-GSTA3 (1:1,000, 16703-1-AP, Proteintech, United States), anti-Foxp3 (1:1,000, GTX107737, GeneTex, United States), anti-TGF-βRI (1:1,000, AF5347, Affinity, United States), anti-TGF-βRII (1:1,000, S0910, Digbio, China), anti-GAPDH (1:5,000, db106, Digbio, China) overnight at 4°C, washed with TBS-T (TBS+0.05% Tween-20), 3 × 5 min, followed by incubation with secondary antibody ( 1:5,000, IRDye 800 CW goat anti-rabbit and IRDye 680 CW goat anti-mouse, LI-COR Biosciences, United Kingdom) for 1 h 30 min at RT. The bands were visualized using Odyssey CLx infrared laser dual colors and the expression of the protein was calculated against GAPDH.

### Isolation of Single-Cell and Fluorescence-Activated Cell Sorting (FACS) Analysis

To analyze the Treg cell and dendritic cell, whole colon was cut into small pieces, immersed in Hanks’ balanced salt solution (HBSS, 10% FBS, 100 U/ml penicillin, 100 μg/ml streptomycin and 2 mM EDTA) and incubated at 37°C, 250 rpm for 1 h. The resultant cell suspension was filtered through a 70 µm filter. The remaining tissue was again minced into small pieces and incubated with hank's balanced salt solution (HBSS) (5% fetal bovine serum (FBS), 100 U/ml penicillin, 100 μg/ml streptomycin, and 1 mg/ml type IV collagenase) for 30 min at 37°C, 250 rpm(Mei et al., 2020). For mesenteric lymph node and spleen, tissue were ground to isolate single cells and treated with RBC lysis buffer to remove red blood cell (RBC) from cell suspension (Salem et al., 2019). The cell suspension was then passed through 70 µm filter and centrifuged for 10 min at 1,500 rpm at 4°C. The pellet was washed with FACS buffer (PBS, 0.1% BSA) and cell density was adjusted to 10^5^/100 ul. The cells were then stained with monoclonal antibody of PE anti-mouse/human CD11b, APC anti-mouse CD11c, APC anti-mouse CD25, FITC anti-mouse I-Ad, or APC anti-mouse CD25, PE anti-mouse FOXP3, FITC anti-mouse CD4, and determined by flowcytometer **(**CytoFLEX, Beckman Coulter, Suzhou, China) for each sample. The percentage of total DC cells, mature DC cells, CD4 + FOXP3 + cells, and CD4 + CD25 + cells were analyzed by CytExpert software (version 12, Beckman).

### Statistical Analysis

Analyzed results are presented as mean ± standard error mean (SEM). Statistical differences were analyzed with GraphpadPrism5 software (Graphpad Software, SanDiego, CA, United States) using ANOVA analysis followed by Bonferroni post-hoc test between selected groups. *p* values lower than 0.05 were considered to be statistically significant.

## Results

### PF-04447943 Mitigates DSS-Induced Colitis in a cGMP Dependent Manner

**FIGURE 1:**
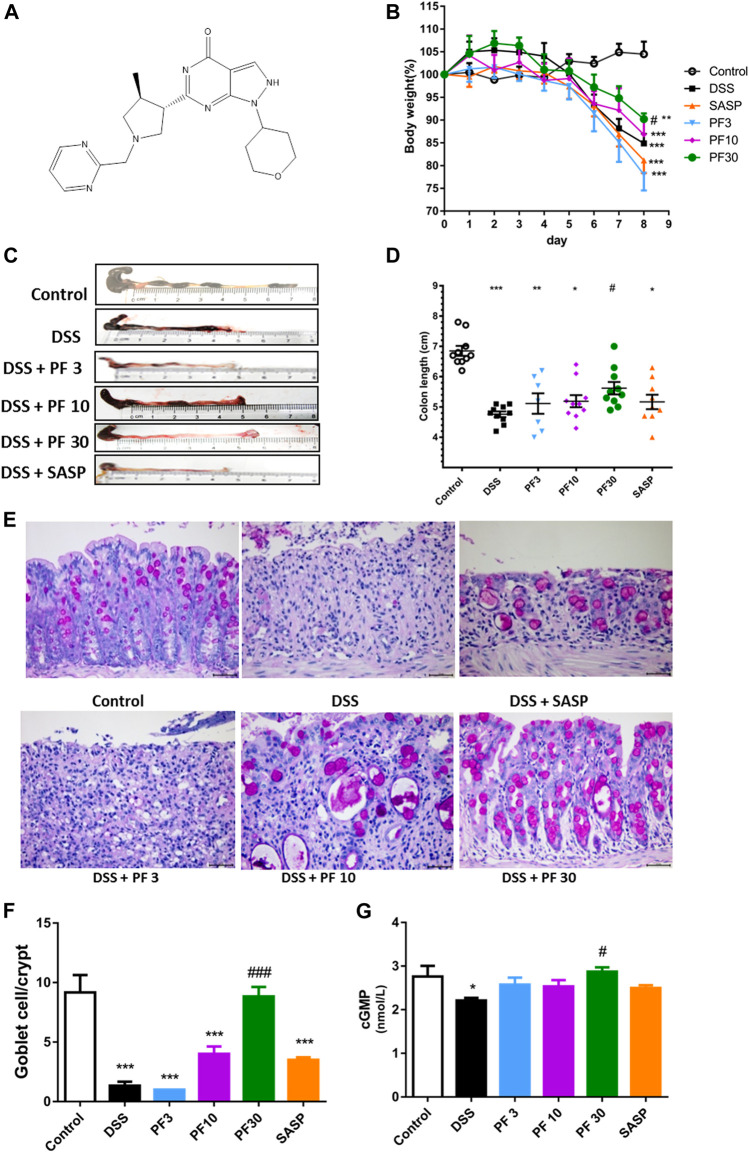
Effect of PF-04447943 on DSS-induced colitis model. Mice were randomly divided into six groups as described in the material and methods section. Control mice received deionized water, DSS-treated mice received (3% DSS in deionized water), co-treatment mice received PF-04447943 at 3, 10, and 30 mg/kg body weight, and sulfasalazine (SASP) at 0.5 gm/kg for 7 days. **(A)** Structure of PF-04447943 drawn by using ChemDraw 15. **(B)** Bodyweight of mice was measured in each group (*n* = 10). **(C)** Microscopic presentation of the colon from each group. **(D)** Quantification of colon length in each group (*n* = 10). **(E)** Representation of goblet cells in the colon by PAS staining. **(F)** Quantification of goblet cells per crypt. **(G)** Quantification of cGMP level in the colon of mice from each group. Data are presented as mean ± standard error mean (SEM) (*n* = 10). Here, ***p < 0.001, **p < 0.01, ^*^ *p* < 0.05 *vs.* control group and ^###^ *p* < 0.001, ^##^ *p* < 0.01, ^#^ *p* < 0.05 vs. DSS-treated group.

To investigate the effect of PF-04447943 on the colitis model, mice were treated with PF-04447943 (Figure 1A) at three different doses by oral gavage. DSS caused a significant body weight loss and shortened the colon length, which were reversed by PF-04447943 in a dose-dependent manner (Figures 1B–D). The histological examination revealed that DSS induced severe mucosal damage, loss of goblet cell, and crypt distortion. The co-treatment of PF-04447943 significantly attenuated the aberrant histopathology and improved the number of goblet cells in a cGMP-dependent manner (Figures 1E–G). However, regarding aforementioned indices of colitis, no significant difference had been observed between mice that received DSS only and co-treated with SASP.

### PF-04447943 Suppresses Oxidative Stress by Inducing Nrf2 and ERK Phosphorylation

**FIGURE 2:**
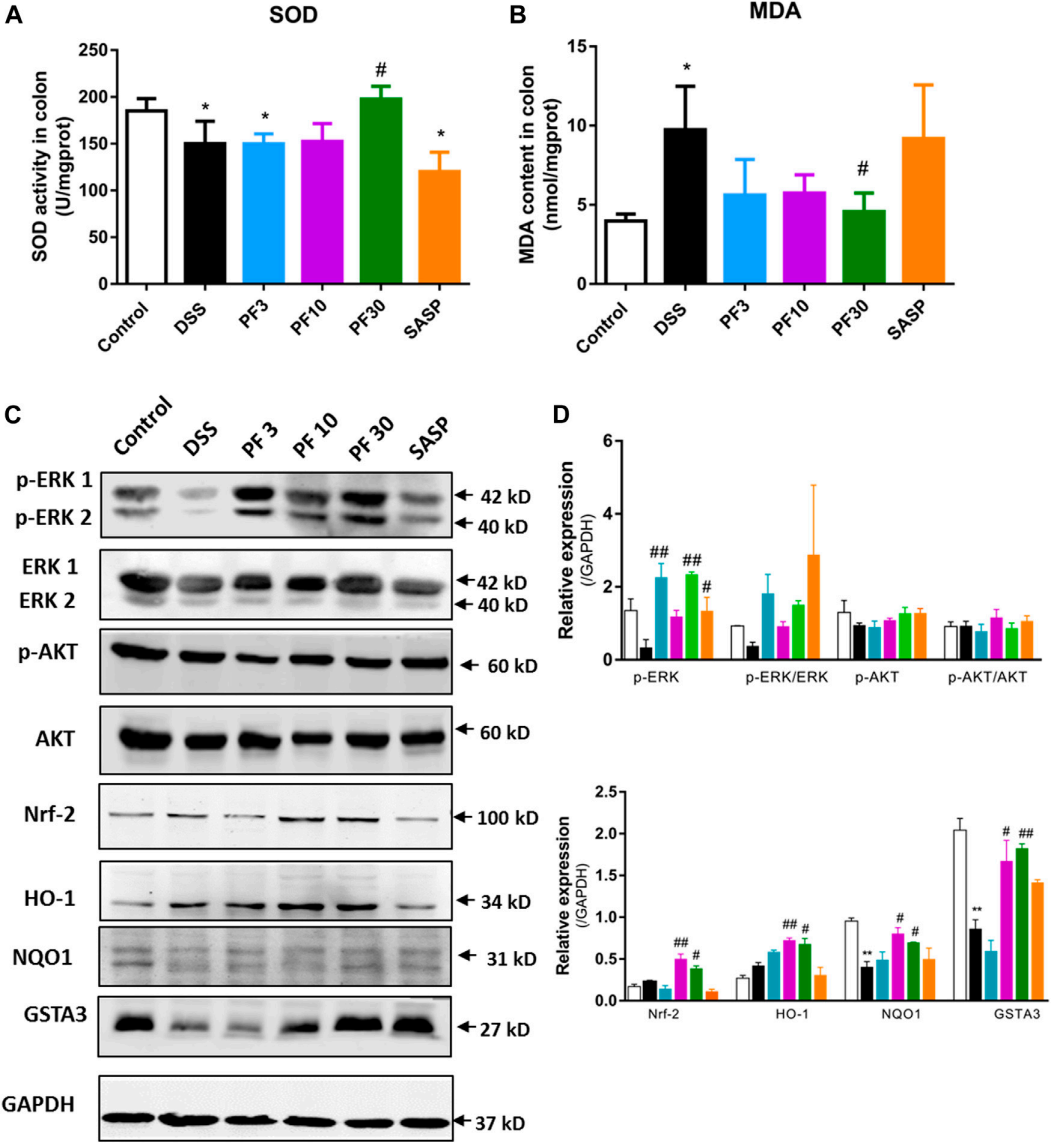
PF-04447943 suppressed oxidative stress inducing Nrf-2 activation against DSS induced colitis mice. **(A)–(B)** The level of SOD and MDA were measured in colon homogenates. **(C)** The expression of *p*-ERK, ERK, p-AKT, AKT, Nrf-2, HO-1, NQO1, and GSTA3 in the colon was assessed by western blot analysis. **(D)–(E)** Relative protein expression of p-ERK, p-AKT, Nrf-2, HO-1, NQO1, and GSTA3, respectively. Data are presented as mean ± standard error mean (SEM) (*n* = 2–5). Here, ***p < 0.001, **p < 0.01, ^*^ *p* < 0.05 vs. control group and ^###^ *p* < 0.001, ^##^ *p* < 0.01, ^#^ *p* < 0.05 vs. DSS-treated group.

To assess the role of oxidative stress, the oxidative stress parameter MDA and level of antioxidant SOD were evaluated. Compared with control, a sharp increase of MDA and decrease of SOD were observed in DSS-treated mice, which were reversed by PF-04447943 at 30 mg/kg BW (Figures 2A,B). These findings suggest that PF-04447943 attenuated DSS-induced colitis by suppressing oxidative stress. But when compared with DSS-treated mice, no remarkable changes had been observed in SASP co-treated mice. To furtherly investigate the mechanism of PF-04447943, we determined the Nrf2 signaling pathway. Results showed that DSS treatment notably downregulate Nrf-2 and its downstream cytoprotective gene, such as heme oxygenase-1(HO-1), NAD(P)H quinone dehydrogenase 1 (NQO1) and glutathione s-transferase α3 (GSTA3) (Figure 2). PF-04447943 co-treatment significantly upregulated the expression of Nrf-2 and its downstream gene. To induce the expression of the Nrf-2 downstream gene, the translocation of Nrf-2 into the nucleus to bind with antioxidant response element (ARE) is essential, which is influenced by phosphorylation of ERK and AKT (Bai et al., 2019). In Figure 2, the results showed that DSS induced a significant decrease in ERK phosphorylation, while the AKT was unaffected. In contrast, compared with DSS-treated group, PF-04447943 remarkably induced the activation of ERK without hindrance to AKT, while no notable changes had been recorded in SASP (Figures 2C–E). These results suggested that the attenuation of DSS-induced oxidative stress was due to ERK-mediated Nrf-2 activation.

### PF-04447943 Attenuates Inflammation Suppressing Cytokines and NF-κB, STAT3 in the Colon and Spleen

**FIGURE 3:**
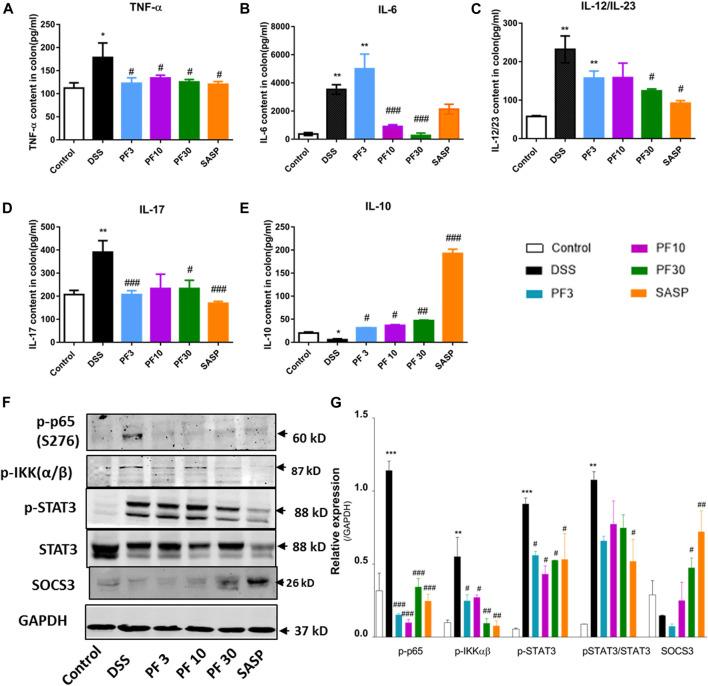
PF-04447943 prohibited inflammation regulating cytokines secretion and suppressing p65, STAT3 activation in DSS-induced colitis mice. **(A)** TNF-α **(B)** IL-6 **(C)** IL-12/IL-23 **(D)** IL-17 **(E)** IL-10 content in colon homogenate were quantified by ELISA assay. **(F)** Expression of p-p65 (S276), *p*-IKKα/β, p-STAT3 (Y705), STAT3, and SOCS3 in colon determined by western blot analysis. **(G)** Relative protein expression of p-p65, p-IKKα/β, p-STAT3, and SOCS3, successively. Data are presented as mean ± standard error mean (SEM)(*n* = 2–5). Here, ***p < 0.001, **p < 0.01, ^*^ *p* < 0.05 vs. control group and ^###^ *p* < 0.001, ^##^ *p* < 0.01, ^#^ *p* < 0.05 vs. DSS-treated group.

To investigate the magnitude of inflammation, specific cytokines, such as TNF-α, IL-6, IL-12/23, IL-17, IL-10, transcription factor p-65, its activator inhibitor of NF-κB kinase α/β (IKKα/β), and STAT3 were assessed in the colon (Figure 3). As expected, a significant elevation of inflammatory cytokines, TNF-α, IL-6, IL-12/23, IL-17, and downregulation of anti-inflammatory IL-10 were recorded in DSS-treated group, while PF-04447943 or SASP notably suppressed the inflammatory cytokines secretion (Figures 3A–E). Besides, PF-04447943, and SASP significantly increased IL-10 secretion. To characterize the pathway, we investigated the expression of p-p65, *p*-IKKαβ, *p*-STAT3, and SOCS3 in the colon. In comparison with control, DSS treatment increased phosphorylation of p65, IKKαβ, and STAT3 at Ser 276, Ser176/180, and Tyr 705, accordingly and decreased SOCS3 expression (Figures 3F,G). In contrast, PF-04447943 and SASP decreased the phosphorylation in a dose-response manner. Meanwhile, SASP significantly decreased the STAT3 and increased SOCS3 in the colon. In the *s*pleen, the STAT3 expression was significantly increased following DSS treatment, which was suppressed by PF-04447943 or SASP, meanwhile, PF-04447943 or SASP upregulated SOCS3 and Foxp3 expression (Supplementary Figure S2). These results reflected the probable anti-inflammatory role of PF-04447943 and SASP was due to, in part, inhibiting p65 and STAT3 phosphorylation, and increasing SOCS3 and Foxp3 expression.

### PF-04447943 Attenuates Inflammasome Activation

**FIGURE 4:**
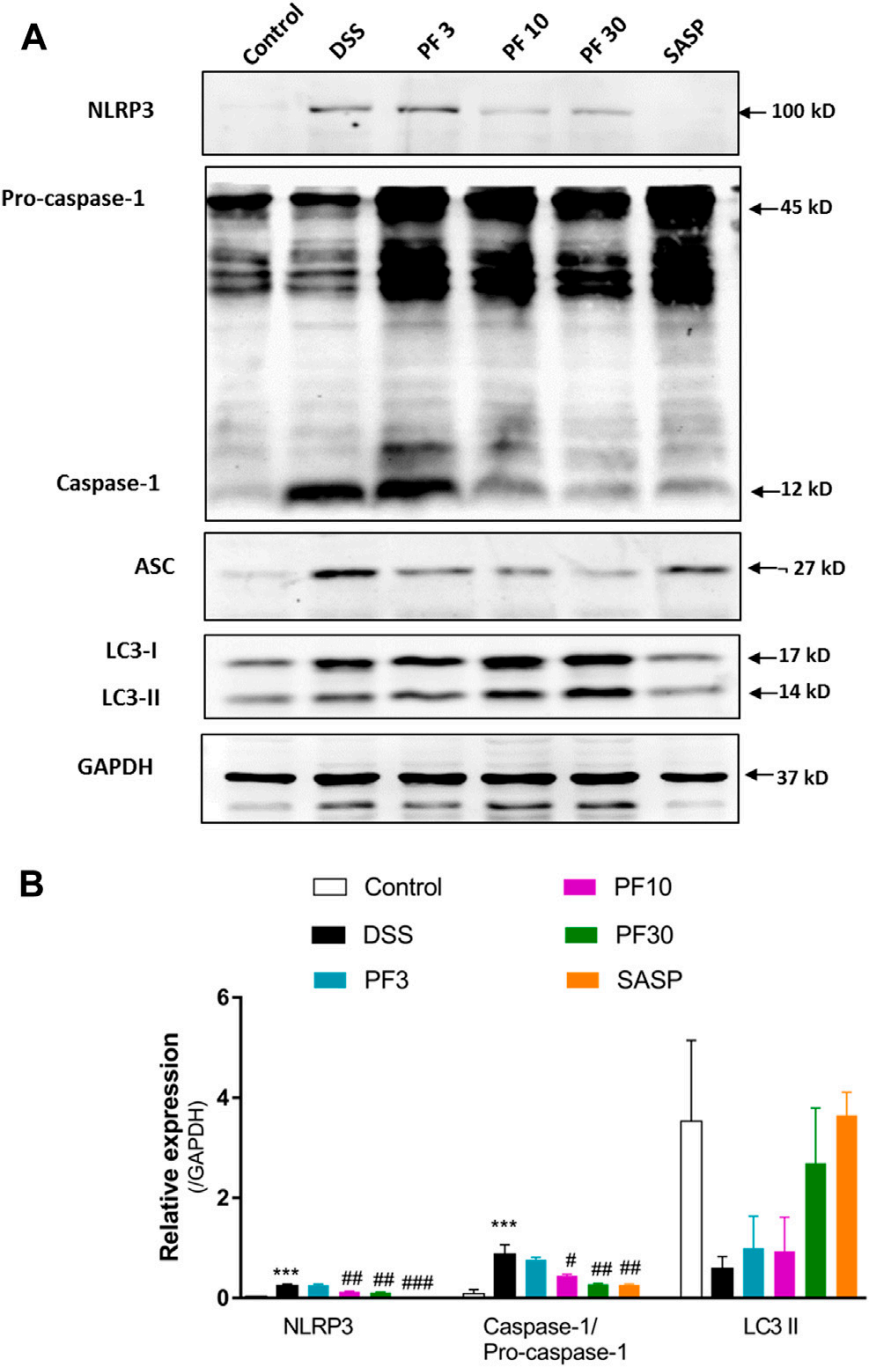
PF-04447943 prohibited inflammasome activation in DSS-induced experimental colitis **(A)** Western blot analysis NLRP3, caspase-1, ASC, LC3, and GAPDH in colon homogenate **(B)** Relative expression of NLRP3, caspase-1, ASC, LC3 protein, respectively. Data are presented as mean ± standard error mean (SEM) (n = 2). Here, ***p < 0.001, **p < 0.01, ^*^ *p* < 0.05 vs. control group and ^###^ *p* < 0.001, ^##^ *p* < 0.01, ^#^ *p* < 0.05 vs. DSS-treated group.

To investigate the contribution of the inflammasome, we determined the activation of NLR family pyrin domain containing 3 (NLRP3) and caspase1. Compared with control mice, DSS-treatment induced significant upregulation of NLRP3 and caspase-1, (Figures 4A,B). However, co-treatment with PF-04447943 at 10 and 30 mg/kg BW diminished the inflammasome activity in a dose-response manner, and SASP had similar effect. Given evidence suggested that autophagy activation regulates the inflammasome activity. Autophagy hinders the NLRP3 inflammasome formation by reducing co-localization of the ASC expression and pro-caspases1, therefore prohibits the maturation of IL-1β (Yuan et al., 2018). According to Figure 4, no profound autophagy activity had been observed in DSS treated mice. Meantime, there was no significant LC3II conversion. Therefore, these data suggested that PF-04447943 reduced the inflammasome activation plausibly by NF-κB activity suppression instead of autophagy.

### PF-04447943 Suppresses the Dendritic Cells Count and Their Maturation

**FIGURE 5:**
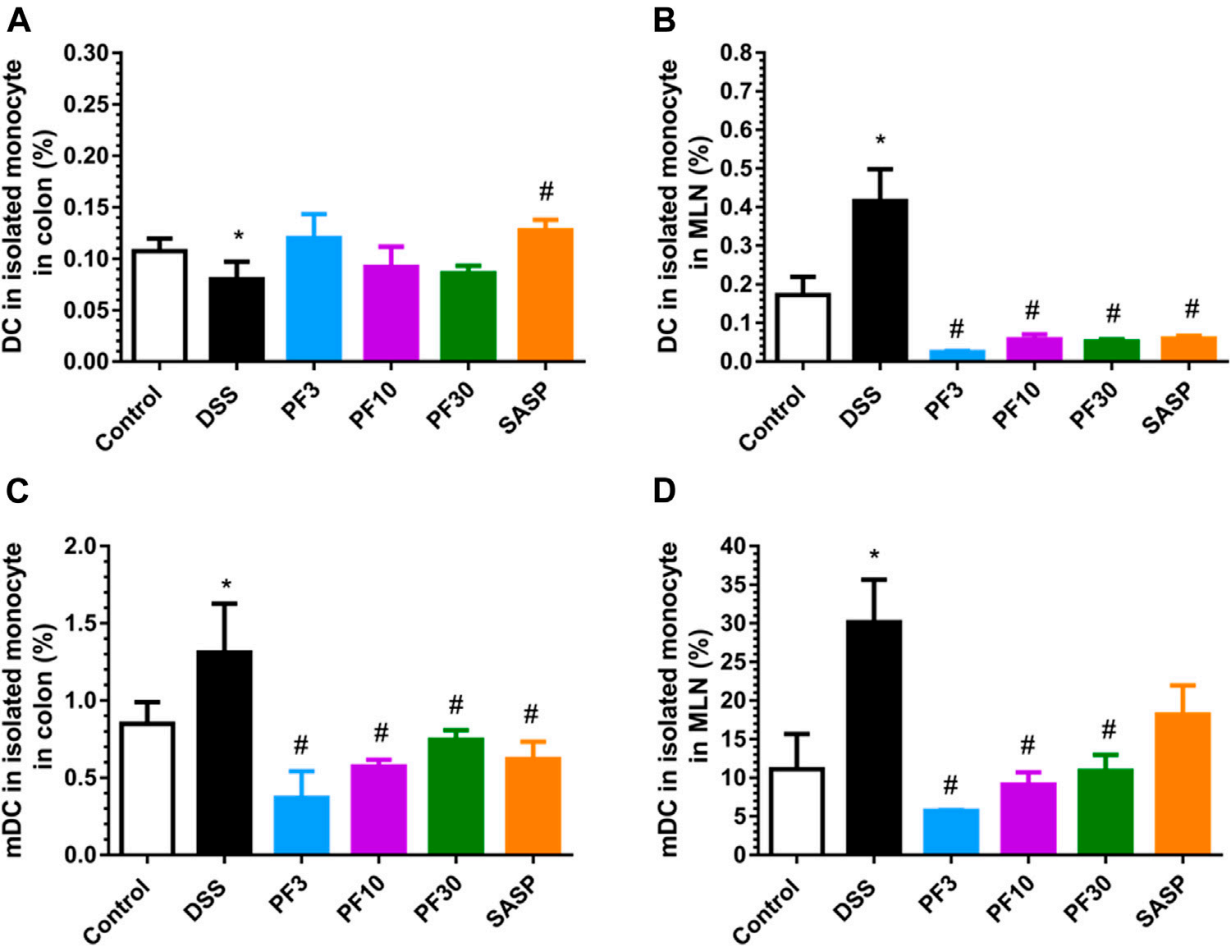
PF-04447943 ameliorated DSS induced colitis suppressing DC and their maturation. **(A)** Percent of total DC cells in isolated monocyte from the colon. **(B)** Percent of total DC cells in isolated monocyte from mesenteric lymph node (MLN). **(C)** Percent of mature DC cells in isolated monocyte from the colon. (**D**) Percent of mature DC cells in isolated monocyte from the MLN. Data are presented as mean ± standard error mean (SEM) (n = 5). Here, ***p < 0.001, **p < 0.01, ^*^ *p* < 0.05 vs*.* control group and ^###^ *p* < 0.001, ^##^ *p* < 0.01, ^#^ *p* < 0.05 vs. DSS-treated group.

DC is the prominent APC which can induce disperse immune response, especially in activation and polarization of T-cells. According to the FACS analysis data (Figure 5), compared with control DSS caused a profound increase of total DC and its mature form (mDC) in mesenteric lymph node (MLN). In contrast, the number of DC were increased in the colon in comparison with control (Figure 5A), but the percentage of mDC was decreased (Figure 5C). However, the PF-04447943 and SASP notably reduced the total DC counts (Figure 5B) along with the mature one in MLN (Figure 5D) except for SASP. These data suggested that DSS-induced the DC activation in the MLN, whereas PF-04447943 or SASP reduced the mDC cells number. In the colon, the total DC number remained same following of PF-04447943.

### PF-04447943 Alters T Cells Polarization by Upregulating the Expression of Forkhead box P3 (Foxp3) in the Colon

**FIGURE 6:**
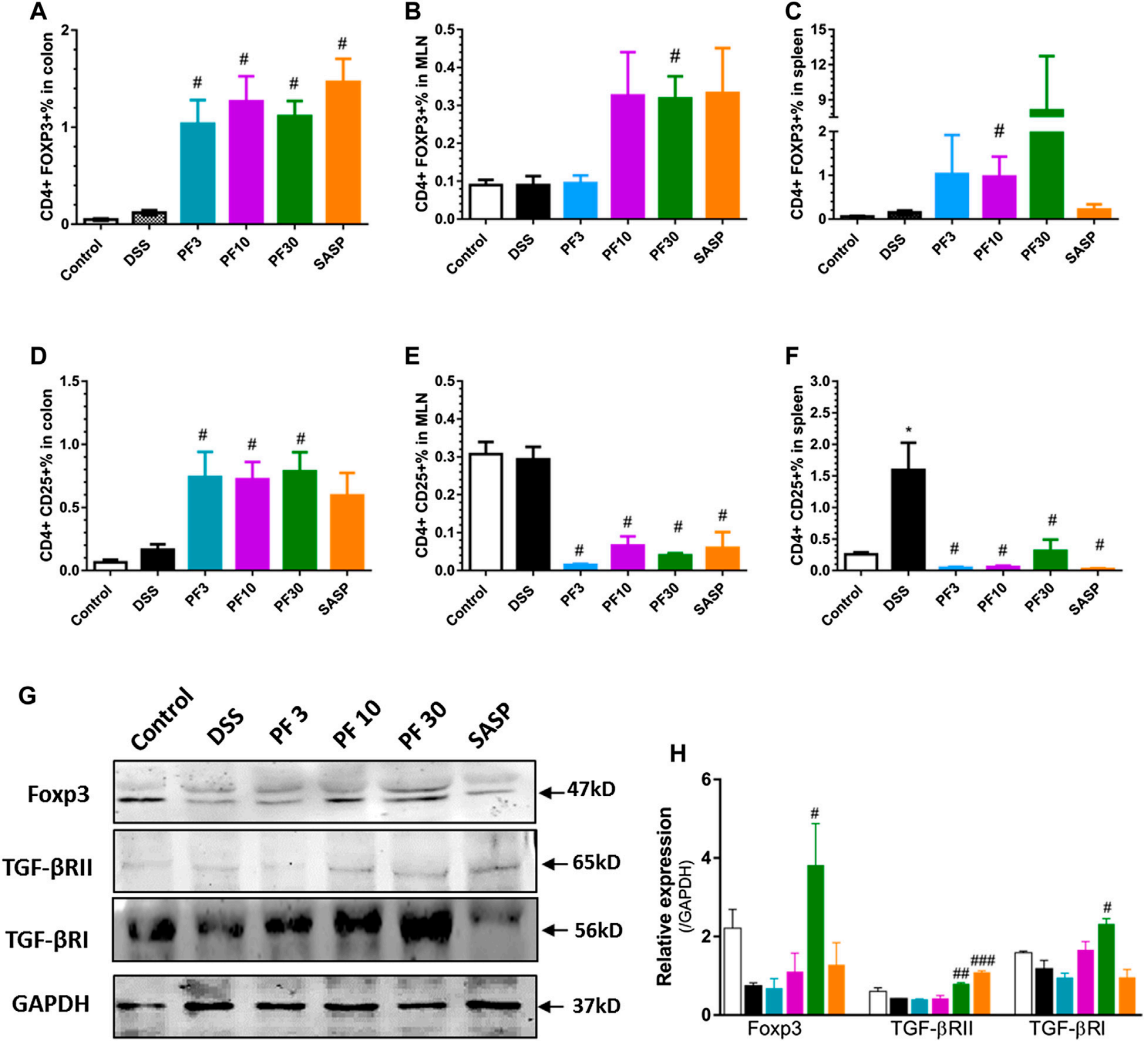
PF-04447943 upregulated Treg cells in DSS induced colitis. **(A-C)** Percent of CD4^+^Foxp3^+^ cells in the colon **(A)**, MLN **(B)** and spleen **(C)**, respectively. **(D-F)** Percent of CD4^+^CD25^+^ cells in the colon **(D)**, MLN **(E)**, and spleen **(F)**, respectively. **(G)** Expression of Foxp3, TGFβRII, TGFβRI, and GAPDH in colon assessed by western blot. **(H)** Relative expression of Foxp3, TGFβRII and TGFβRI, successively. Data are presented as mean ± standard error mean (SEM) (*n* = 2–5). Here, ***p < 0.001, **p < 0.01, ^*^ *p* < 0.05 vs. control group and ^###^ *p* < 0.001, ^##^ *p* < 0.01, ^#^ *p* < 0.05 vs. DSS-treated group.

To assess T-cells polarization, we evaluated the Treg cells in the spleen, MLN as well as in colon by FACS analysis. As shown in Figure 6, the percentage of CD4^+^CD25^+^, CD4^+^FOXP3^+^ cells (Treg cells) in the colon of control and DSS-treated mice had no differences. Expectedly, the PF- 04447943 and co-treatment remarkably increased the percent of Treg cells in colon (Figures 6A,D). This phenomenon might reflect the polarization of Th17/Treg cells to Treg/Th17 cells followed by enhanced expansion of Tregs in colitis. Intriguingly, the number of CD4^+^CD25 ^+^ cells was increased in the spleen and MLN of DSS-treated mice (Figures 6E,F), while the percentage of CD4+FOXP3+ cells was decreased (Figures 6B,C), suggested the absence of Treg cells in DSS-induced colitis. These results described that PF-04447943 helps to maintain the Treg/Th17 cells balance by expanding Treg cells.

To furtherly investigate the mechanism about the upregulation of Treg cells in the colon, we also determined the expression of transcription factor Foxp3, and receptors of transforming growth factor beta (TGFβ) by western blot analysis. The TGFβ bind to the its receptor transforming growth factor beta 1 receptor (TGFβRI) and subsequent phosphorylation events of smad leads to expression of gene including Foxp3 (Fantini et al., 2004). The high expression of Foxp3 suggests the expansion of Treg cells, while blockage of TGFβRI reduces the Treg cells generation and suppresses Treg function (Polanczyk et al., 2019). Figures 6G,H suggested that Foxp3 expression along with TGFβRs decreased in DSS-treated mice. However, PF-04447943 enhanced the expression of Foxp3 and TGFβRs (Figures 6G,H) in a dose-response manner. These data supported the expansion of Treg cell in the colon when PF-04447943 was administered. Nevertheless, SASP failed to induces the expression of TGFβRI and Foxp3.

## Discussion

This study reports that a selective inhibitor of PDE9A, PF-04447943 protects colon against DSS-induced colitis for the first time. In this study, we showed that PF-04447943 attenuates the oxidative stress and inflammation in a dose-dependent manner by suppressing NF-κB, STAT3, inflammasome activation, and activating Nrf2 pathway. Besides, PF-04447943 reverses the imbalance of Treg and Th17 cells induced by the DSS treatment by upregulating the expression of TGFβR1.

Oxidative stress is a well-known pathophysiological mechanism in inflammation-associated diseases including IBD. It brings the damage to the intestine and disrupts the homeostasis of mucosal immunity (Tian et al., 2017). Enhanced oxidative stress and reduced goblet cells were evidenced following DSS treatment (Zhao Z. et al., 2019). To assess oxidative stress, we investigated the MDA, SOD level, and histopathology of the colon. These were attenuated by PF-04447943 in a dose-dependent manner. Consistently, PF-04447943 also protected the integrity of colonic architecture. Transcription factor, Nrf-2 suppresses the oxidative stress by induction of downstream gene such as SOD, NQO1, HO-1 and GSTA3 (Chen and Maltagliati, 2018; Yang et al., 2002). Hwang and colleagues reported apocynin plays the protective role by upregulating Nrf-2 and HO-1 in DSS-induced colitis (Hwang et al., 2019b). Our results suggested the Nrf2 pathway was also activated by PF-04447943 in a dose-dependent manner. In the oxidative stress condition, the upregulation of ERK and AKT show the protective role to induce the expression of cytoprotective gene, such as Nrf2 and HO-1 (Wang et al., 2019). Nevertheless, ERK and AKT are profound for their inflammatory role in UC (Gao et al., 2019; Li et al., 2019). But there are growing evidences to support ERK phosphorylation promote Nrf2 expression in colitis. The gastroprotective drug, rebamipide facilitated the healing via upregulating ERK phosphorylation in TNBS-induced colitis model (Takagi et al., 2011). A recent study also revealed that ERK and AKT induces the Nrf-2 translocation and transactivation to reverse the oxidative stress in colitis (Bai et al., 2019). Regarding this perspective, upregulated ERK phosphorylation with enhanced expression of Nrf-2 and its gene battery by PF-04447943 was confirmed in our current study. But in our study, SASP did not increase the Nrf2 and HO-1, as well as p-ERK and AKT, suggested that SASP and PF-04447943 activates different signaling pathway.

Meanwhile, the oxidative stress and inflammation are closely interconnected to induce pathophysiology of ulcerative colitis (Jeon et al., 2020). p65-NF-κB plays a crucial role in activating inflammasome to propagate the inflammatory microenvironment, and inflammasome mediated Th17 cells differentiation in colitis (Zhao W. et al., 2019) (Zhang et al., 2014). In contrast, autophagy activation suppresses the activity of NLRP3 inflammasome preventing the cleavage of caspase-1 (Cosin-Roger et al., 2017; Liu et al., 2018). Previous study has indicated that NK-κB and NLRP3 inflammasome were activated in DSS-induced UC, which were accompanied by the pro-inflammatory cytokines, such TNF-α, IL-6, and IL-1β (He et al., 2019). In the present study, PF-04447943 attenuates the p65 phosphorylation and NLRP3 activation with decrease of oxidative stress and pro-inflammatory cytokines, but we did not find the autophagy activation, that suggested that the major action of PF-0444793 may be on NF-κB.

In IBD pathogenesis, the role of the transcription factor, STAT3 both in the hematopoietic and non-hematopoietic lineage is essential (Li et al., 2012). IL-6 augments the expression of STAT3 in the colon, thereafter, regulates the polarization of IEC (Serrano et al., 2019). T cell-specific knockout of STAT3 ameliorates DSS-induced colitis by reducing the inflammatory response (Kwon et al., 2018). In our study, we uncovered that DSS induces the upregulation of STAT3 in the colon along with other inflammatory cytokines including IL-6. PF-04447943 downregulated STAT3 activation, probably mediated the suppression in T cells and then reduced the IL-6. Suppressor of cytokine signaling 3 (SOCS3) is a downstream protein of STAT3. The activation of DCs via JAK/STAT/SOCS signaling pathway was reported in DSS induced colitis mice, whereas curcumin suppressed the activation of DCs by reducing STATs expression, enhancing SOCSs expression produce protective effect (Zhao et al., 2016). Coherent to this reports, PF-04447943 also upregulated SOCS3 following the decrease of STAT3, and this employed mechanism may also mediates the DC inhibition. Similar to the colon, PF-04447943 also attenuated the enhanced expression of STAT3 by upregulating the SOCS3 and Foxp3 in the spleen (Supplementary Figure S2).

In the pathogenesis of UC, Treg/Th17 cells imbalance is an important immune mechanisms. Britton and colleagues suggested that Th17-based effector T cell responses exacerbate disease severity in IBD (Britton et al., 2019). Intriguingly, the STAT3 also regulates the differentiation of Th17 cells in colitis, impairs the Treg/Th17 cells balance (Xu et al., 2019). Recent finding reported that DC serves as a potential and efficient APC to regulate the Treg and Th17 cells balance in colitis model (Zheng et al., 2020). Both of the cells are derived from naïve T cells, where Th17 cells secret pro-inflammatory IL-17 predominantly, while Treg cells secrets immunosuppressive including IL-10 and TGF-β (Xu et al., 2015). Previous studies suggested that recovering the Treg/Th17 cells balance and limiting the subsequent secretion of corresponding cytokines ameliorates the extent of colitis (Xu et al., 2015; Yao et al., 2015). In addition, rebamipide, a drug for treatment of UC, also attenuates the inflammation in arthritis and gut by rebalancing the Treg/Th17 cells, suppressing TNF-α, IL-17 level and modulating splenic STAT3 activity (Moon et al., 2014; Min et al., 2016). According to our current findings, PF-04447943 also protects colon from colitis by reversing the Th17/Treg cells balance with suppression of DC and downregulation of IL-17, 1L-12/23, and IL-6. However, an approach to neutralize IL-17 results in aggravation of symptoms of IBD (Wang et al., 2018). We revealed that a decrease in IL-17 level was not due to IL-17 neutralization but upregulation of Treg cells. Herein, upregulation of TGFβRI, TGFβR2 as indices of TGF-β elevation along with increased Treg cell number, and Foxp3 expression in the colon are evidenced in the co-treatment of PF-04447943. However, the upregulation of TGF-β alone in the presence of IL-6 is not capable of differentiating naïve CD4^+^ cells into Treg cells (Bettelli et al., 2006). During naïve condition, TGF-β upregulates the transcription factor, Foxp3, and RORγt that are related to Treg and Th17 cells, respectively (Zhou et al., 2008). Interestingly, ERK could upregulate the expression of TGF-βRs (Hwang et al., 2019a). In the process of Th17 cell differentiation, RORγt upregulates the transcription IL-17 through binding to its promoters (Ichiyama et al., 2008). However, the upregulated Foxp3 binds with RORγt, thus hinder the Th17 development (Zhou et al., 2008). Following the accumulation of IL-6 and IL-12, they activate the STAT3 and STAT4, thus boost up the Th17 developments, upregulate the expression of IL-23R and IL-23R mediated Th17 cell expansion (Lee et al., 2017). Taken together, PF-04447943 suppresses the inflammatory cytokine, Th17 expansion, and increase Treg cells by inhibition of STAT3 and activation of TGFβR. The ELISA assay results also suggested that PF-04447943 elevates the IL-10 level in the colon. Regarding the function of IL-10, it regulates the immune tolerance activity of Treg cells, apart from its anti-inflammatory role against inflammasome (Chaudhry et al., 2011; Zhang et al., 2014).

In conclusion, this study suggested that PF-04447943 ameliorates DSS-induced colitis by prohibiting oxidative stress and inflammation by activating Nrf-2 and modulating NF-kB, STAT3, and inflammasome activity. Importantly, PF-04447943 also amends the inflammatory microenvironment by rebalancing the Treg/Th17cells through upregulating Foxp3 expression and intervening in the accumulation of dendritic cell and their maturation. Therefore, PF-04447943 could be regarded for further study as a possible candidate of IBD therapy based on Treg/Th17 cells proportion to regulate the mucosal immunity. And our study may open up a new avenue for PDE9A inhibitor.

## Data Availability

The raw data supporting the conclusions of this article will be made available by corresponding authors on reasonable request.

## Supplementary Material

FIGURE S1 The mRNA expression of PDE superfamily in the colon of DSS induced UC model in female C57 mice (10 weeks). Data are presented as mean ± standard error mean (SEM) (n=3).

FIGURE S2 PF-04447943 suppressed inflammation suppressing STAT3 and upregulated Foxp3 expression in spleen. **(A)** Expression of p-STAT3 (Y705), SOC3, Foxp3, and GAPDH in spleen determined by western blot. **(B)** Relative expression of p-STAT3 (Y705), SOC3, and Foxp3, respectively. Data are presented as mean ± standard error mean (SEM) (n=2). Here, ****p* < 0.001, ***p* < 0.01, ^*^ *p* < 0.05 *vs*. control group and ^###^ *p* < 0.001, ^##^ *p* < 0.01, ^#^ *p* < 0.05 *vs*. DSS-treated group.

## Glossary

IBD: inflammatory bowel disease
UC: ulcerative colitis
cGMP: cyclic guanosine monophosphate
cAMP: cyclic adenosine monophosphate
PDEs: cyclic nucleotide phosphodiesterases
PDE9A: phosphodiesterase 9A
DSS: dextran sulfate sodium
SOD: superoxide dismutase
MDA: malondialdehyde
ELISA: enzyme-linked immunosorbent assay
NF-kB: nuclear factor kappa B
STAT3: signal transducer and activator of transcription 3
SOCS3: suppressor of cytokine signaling 3, Treg cells-regulatory T cells, Th17 cells- T helper 17 cell
ERK: extracellular signal-regulated kinase
Nrf-2: nuclear factor erythroid 2–related factor 2
HO-1: heme oxygenase-1
GSTA3: glutathione s-transferase α3
NQO1: NAD(P)H quinone dehydrogenase 1
DC: dendritic cells
TNF-α: tumour necrosis factor alpha
IL-1β: interleukin 1 beta
IL-6: interleukin 6
IL-33: interleukin 33
IL-10: interleukin 10
RORγt: retinoic acid-related orphan receptor gamma t
ROS: reactive oxygen species
APC: antigen presenting cell
JNK- c-: Jun N-terminal kinase
HBSS: hank's balanced salt solution
FBS: fetal bovine serum
FACS: fluorescence-activated cell sorting
NLRP3: NLR family pyrin domain containing 3
MLN: mesenteric lymph node
Foxp3: forkhead box P3
TGFβR: transforming growth factor beta receptor
TGFβ: transforming growth factor beta
TGFβRI: transforming growth factor beta 1 receptor
TGFβRII: transforming growth factor beta 2 receptor
