# Erratum: PDE9 inhibitor PF-04447943 attenuates DSS-induced colitis by suppressing oxidative stress, inflammation, and regulating T-cell polarization

**DOI:** 10.3389/fphar.2024.1393029

**Published:** 2024-03-11

**Authors:** Frontiers Production Office

**Affiliations:** Frontiers Media SA, Lausanne, Switzerland

**Keywords:** ulcerative colitis, PF-04447943, inflammasome, Treg/Th17 cell balance, PDE9A, oxidative stress

Due to a production error, a footnote (†) to the author list was incomplete and was also incorrectly not applied to the author Mohammad Nasiruddin Rana. The correct author list and footnote are as follows:

Mohammad Nasiruddin Rana^1†^, Jie Lu^2†^, Enfu Xue^1†^, Jingjing Ruan^1†^, Yuting Liu^1†^, Lejun Zhang^1^, Rana Dhar^1^, Yajun Li^1^, Zhengqiang Hu^1^, Jie Zhou^3^, Wangqian Ma^4*^ and Huifang Tang^1*^

^†^ These authors have contributed equally to this work and share first authorship

The publisher apologizes for this mistake. The original version of this article has been updated.

