# Supplementary figures and images for: PDE9 Inhibitor PF-04447943 Attenuates DSS-Induced Colitis by Suppressing Oxidative Stress, Inflammation, and Regulating T-Cell Polarization
Source: Front Pharmacol. 2021 Apr 8;12:643215. doi: 10.3389/fphar.2021.643215 (PMC8098793; doi:10.3389/fphar.2021.643215)

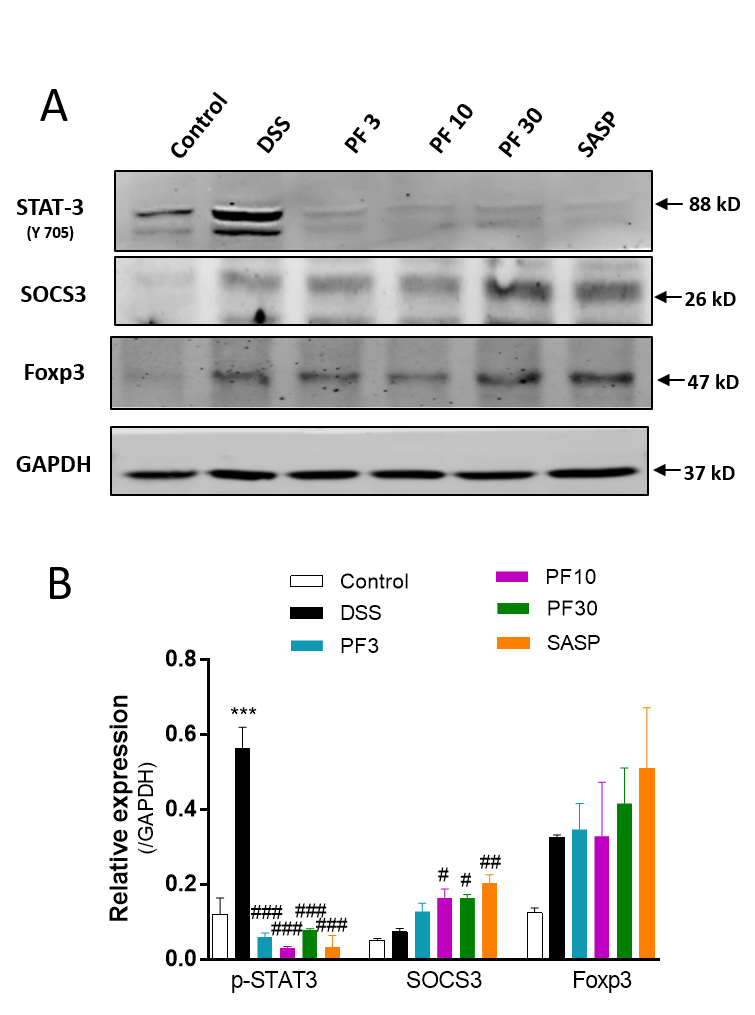

Supplement: Supplementary file 1 [file Image2.tif]

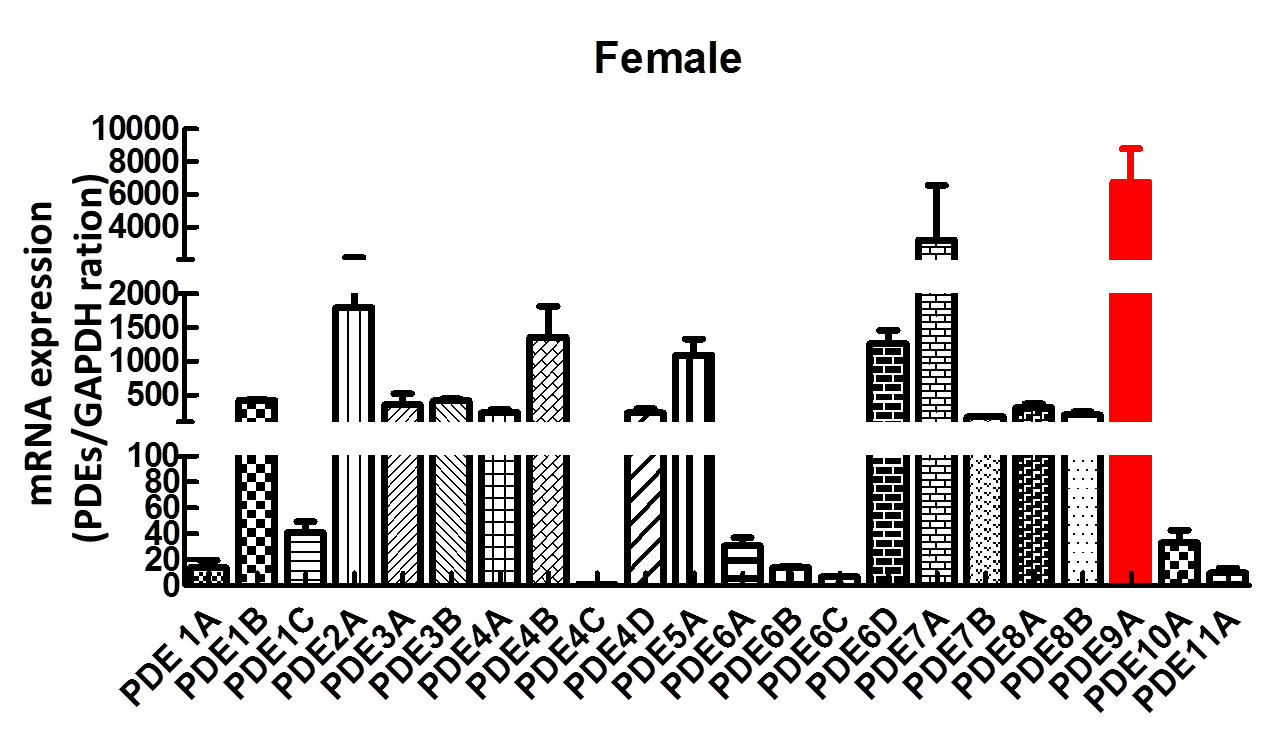

Supplement: Supplementary file 2 [file Image1.tif]
